# Interfacial Adhesion of Thick NiTi Coating on Substrate Stainless Steel

**DOI:** 10.3390/ma15238598

**Published:** 2022-12-02

**Authors:** Sneha Samal, Jaromír Kopeček, Petr Šittner

**Affiliations:** FZU—Institute of Physics of Czech Academy of Science, Prague 8, Na Slovance 1999/2, 18221 Prague, Czech Republic

**Keywords:** NiTi, thick layer, plasma process, stainless steel substrate

## Abstract

Interfacial adhesion of thick NiTi coating on substrate stainless steel is investigated here. NiTi coating was deposited on the substrate by using the thermal plasma spraying method. Deposition of NiTi coating was carried out by using various levels of input power under an Ar atmosphere. Multiple coating layers were deposited on the stainless steel surface for a specific thickness. The cross-section of the plasma-sprayed samples were prepared and characterized by using various techniques. The hardness of the coating layers on the surface and cross-section was examined. The thickness of the coating increased with the increase in power. No cracks were detected in the interface for the NiTi coating deposited at 12 kW power. However minor pores were observed at some regions along the interface at the sample prepared at 9 kW power. A good-quality coating layer was formed at the interface of the substrate. Primary phases of austenite and martensite were confirmed from the EBSD and XRD investigations. There was the presence of intermetallic and oxide phases in the coating layers. A less heat-affected zone of 10 µm of along the interface was confirmed without any diffusion of elements from the substrate to the coating layers. There was homogenous distribution elemental composition of Ni and Ti throughout the coating layers.

## 1. Introduction

The lifetime and durability of the shape memory alloy in various aggressive environments need to focus on the attention of the surface quality and its exposure to various environmental conditions. The improved surface of the material could be achieved by coating it in various ways. The coating protects the inner core of the material from outer exposure, environmental conditions, and corrosion. NiTi coating is applied as an efficient functional coating in improving the surface features. Improved surface quality is one of the crucial parameters for materials to contribute toward lifetime uses. The thermal plasma arc process was widely used for the surface treatment of materials [1,2,3]. Thermal plasma technology is considered one of the potential ways to improve the surface properties of the materials. The thermal plasma process provides a higher deposition rate, a faster process with a one-step approach for coating materials. Argon gas maintains neutrality in the plasma chamber to avoid unnecessary reactions from contaminations within the coating. Nickel–titanium (NiTi) alloy coating is considered a functional coating with shape memory properties that add benefits to surface properties [4,5]. NiTi alloy has several applications from engineering to medical applications. One of the common uses of the coating on the material against erosion and corrosion [6,7]. The performance of NiTi coating against erosion and corrosion stands out as being more outstanding than other conventional materials used for coating [8]. The NiTi coating could withstand high temperatures with the capability of displaying high oxidation resistance. Simultaneously, thermo-mechanical properties of NiTi added an advantage in increasing the wear resistance due to its pseudo-elasticity nature. Stainless steel displays a low hardness of 129 HV that contributes towards low cavitation erosion resistance. The cavitation and erosion properties of stainless steel are improved by various surface treatments such as laser processing using different materials as a coating layer. However, this improvement was accompanied by weaker corrosion resistance in the material. Improving overall properties such as erosion–corrosion resistance in the material NiTi powder is considered a potential material for improved surface properties [9,10,11]. There are various technologies such as welding and high-temperature brazing involved in coating filler materials on the steel substrate [12,13,14,15]. However, there are obstacles of various heat-affected zones from the substrate to the coating layer in these processes that modify the microstructure of the materials. These obstacles could be overcome by plasma spraying technologies, as the process involves cooling the substrate during the process of deposition. The subsequent cooling and deposition process occurs in phases, and as a result, there is no change in the temperature zone occurring in the substrate. Plasma spraying has been used in various materials for coating purposes including NiTi powders [16,17,18]. Interfacial adhesion of functional coating has a significant influence on composite behavior in various sensitive environmental applications [19]. Materials used in various applications face the challenge of withstanding erosion and corrosion in extreme environmental conditions. The materials with enhanced surface features could be achieved by surface modification technique by implementing functional coating on the material through various compositions and improved surface properties. Functional coating of NiTi has attracted the coating fields due to the functional behavior of shape memory effect (SME) and a superelasticity (SE) nature with good wear and excellent corrosion resistance properties [20]. NiTi is considered a corrosion and wear-resistant coating due to its SE, SME, and high damping capacities [21]. NiTi coating also has been proven towards the cavitation erosion layer with high resistance. The chemical inertness of NiTi arises from the barrier protective layer of NiTi that forms a protective oxide layer with direct contact with the environment. Stainless steels are widely used materials in surgical tools and medical equipment [22]. They can be rolled into sheets, plates, bars, and wires and can be used for many purposes from engineering materials to infrastructure in aggressive environments [23]. However, the lifetime and durability of the material are improved by coating layers that protect against erosion and corrosion [24]. In this scenario, functional coating materials such as NiTi coating are considered to be one of the potential materials that could protect against corrosion and erosion to the base material. 

Various surface treatment methods have emerged to improve the surface properties of materials either by coating or surface annealing. As the thermal plasma process provides a higher deposition rate without any contamination, as an easy, one-step, quick process, the use of this technology is increasing faster. This process operates in a neutral environment with a cooling effect on the substrate that may influence the heat-affected zone in the substrate less. 

NiTi powder was deposited on the surface of an austenitic stainless steel (AISI 304) plate by using plasma spraying. The thick coatings were deposited on multiple layers using different powers using Ar gas in the plasma chamber. The surface image of the interface layer from coating adhesion to the substrate, phase formation, mechanical behavior, and hardness was examined by using various characterization processes. In order to explore the nature of the adhesion of NiTi coating with stainless steel substrate, the powders of NiTi were chosen for coating to investigate whether the NiTi phases can occur in thermal spray coatings. The role of intermetallic phases towards hardness was interpreted. We also investigated the connection between the interfacial microscale reactions, splat substrate bonding, and adhesion between interlayers throughout coating thickness.

## 2. Experimental Methods

The thick coating of NiTi was deposited on an austenitic stainless steel (AISI 304) plate by using a radio frequency inductively coupled plasma (RF-ICP) facility from the Institute of Plasma physics (IPP), Prague. The operating experimental parameters of the spraying method are represented in Table 1. The dimensions of the substrate of 60 × 20 × 3 mm were chosen. The substrate was cleaned with acetone in order to remove dust and impurities before coating. Table 1 represents the parameters for spraying NiTi powder for the preparation of samples. Table 1 displays the chemical composition of NiTi powder and substrate. 

**Figure 1:**
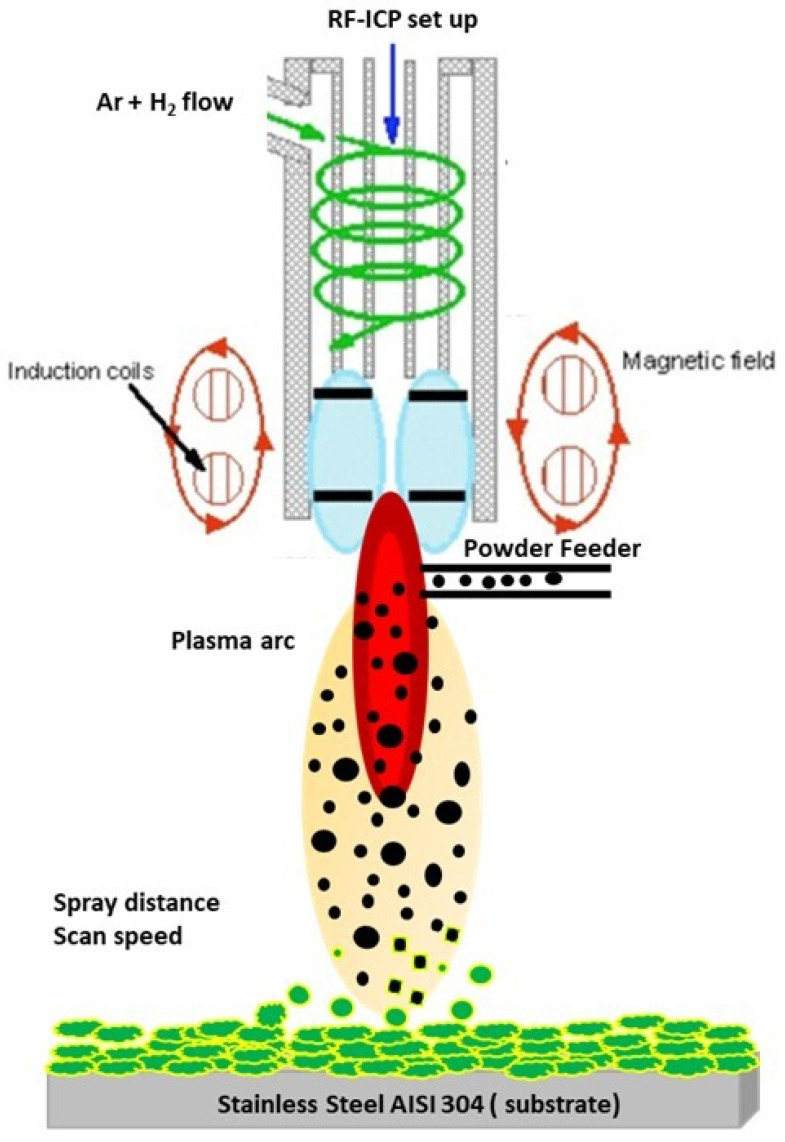
Impact of melting particles on the substrate through the plasma arc created a multi-coating layer’s structure.

The plasma source was used in the RF-ICP chamber as the heat source for melting NiTi particles and impact on the substrate for deposition. The impact of powder particles through the plasma arc that leads to the deposition on the stainless steel substrate (Italinox, Prague, Czech Republic) is shown in the schematic diagram in Figure 1. The dimensions of the substrate were 60.6 × 20 × 3 mm^3^ [11]. The powder particles were inserted into the plasma arc by the powder feeder using various powder feed rates that allow particles to travel through various zones of the plasma arc. The inner and outer regions of the plasma zone distributed various temperature zones that allow for the melting of particles. The melting particles consider a “fully melted or partially melted splat” that impacts the substrate and forms a layer of coating. The multi-pass of the coating layers forms a certain thickness. 

The metallographic examination of a multilayer-coated surface on the substrate involved preparing a sample by cutting from the bulk cross-section-wise using an Electric Discharge machine. The samples were polished with up to 1 µm diamond paste. The microstructural examination was carried out on the samples electrolytically etched in a solution of (HF:HNO_3_:H_2_O) etchant. The microstructure of the NiTi powders and plasma-sprayed samples was investigated by an optical microscope (OM) and scanning electron microscope (SEM, Tescan FERA 3 (Tescan, Brno, Czech Republic)). The images were investigated by both modes of observation using secondary electrons and backscattered electrons. Energy-dispersive X-ray spectroscopy (EDS) was carried out using the EDAX system (EDAX, Ametek Inc., Mahwah, NJ, USA) with an Octane Super 60 mm^2^ detector to determine the chemical composition from substrate towards coating. The phase and grain orientation distribution analyses were performed using the electron back-scattered diffraction method (EBSD) using the EDAX DigiView V camera and system (EDAX, Ametek Inc., Mahwah, NJ, USA). A voltage of 20 kV, a current of 1–2 nA, and a working distance of 15 mm were chosen for analysis. The EBSD sample followed the procedure as samples were hot-mounted in conductive Bakelite and were metallographically prepared by the modified procedure for nickel (ASTM C-56 recipe). The colloidal silica was used in the last polishing step, and the surface was finished by etching in Kroll’s reagent. Grain orientation and phase map of the sintered NiTi alloys were performed by the electron backscatter method. The phase analysis of the coating layers was detected at room temperature with an X’Pert PRO θ-θ powder diffractometer using Bragg–Brentano geometry at 40 mA and 35 kV with CoKα radiation (average wavelength λ = 0.1790 nm), a focus-slit distance of 100 mm, and a goniometer radius of 240 mm. The data were measured in the 2θ range of 20–120°, using a step size of 0.013°, a scan step time of 1.4 s, and a fixed divergence slit size of 0.5°. The phase transformation temperatures of coating layers were carried out by differential scanning calorimetry (DSC 25, TA Instruments, New Castle, DE, USA) at a heating and cooling rate of 5 °C/min in the temperature range of −50 to +150 °C in a nitrogen environment inside the sample chamber. 

Plasma spraying has emerged as one of the potential routes to deposit thick coating layers on the structural material. However, the suitability of reliable methodology could be more widely accepted by assessing the shape memory properties still maintained by coating layers. These properties could only be tested by standalone samples and samples with 0.1 mm of substrate attached. Thermo-mechanical analysis was carried out on the sample in bending mode with a static load of 100 mN from −50 to +150 °C in the temperature cycle. The microhardness of the coating on the surface and cross-section was investigated by a Vickers hardness tester for a force of 1.961 N for a duration of 10 s. 

## 3. Results and Discussion

### 3.1. Surface Features of NiTi Powders, Coating Surface by OM and SEM

**Figure 2:**
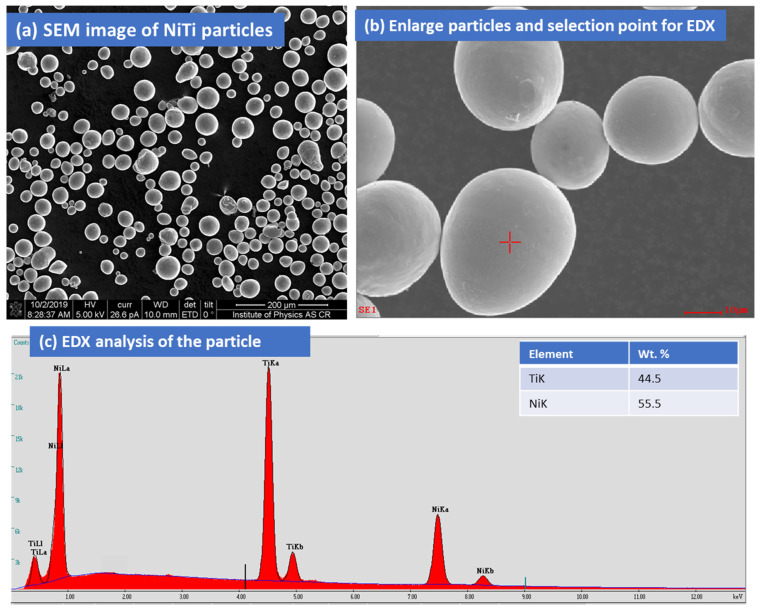
(**a**,**b**) Surface image of NiTi particles. (**c**) EDX analysis of the particle and element composition in wt % (inset).

**Figure 3:**
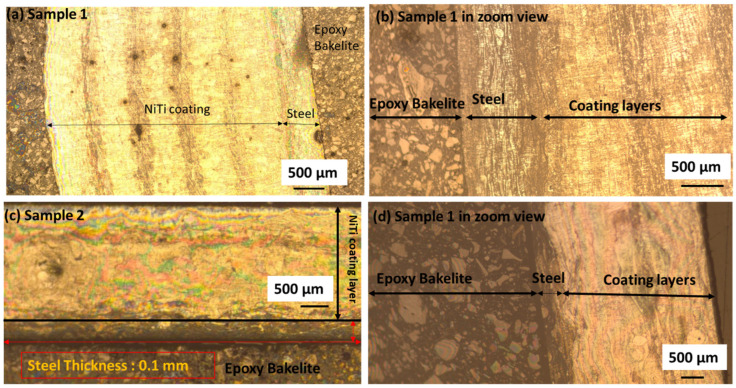
(**a**–**d**) Samples 1 and 2 with substrate steel and coating layers showing adhesion of the surface.

Figure 2 represents the Ti_50_Ni_50_ powder particle (purity: 99.5%) that is considered for the thick multilayer coatings on stainless steel. The gas-atomized NiTi powders were purchased from American Elements (AE), MERELEX CORPORATION, LOS ANGELES, CA 90024, USA. The elemental composition of the Ni:Ti powders was 50:50 (atom %). NiTi particles are spherical in shape and size (Figure 2a,b). The average particle size of NiTi powder (Avg) was 50 µm. The sizes of the largest particles were in the range of 30–40 µm. EDX analysis revealed the chemical composition of NiTi powders, as shown in Figure 2c. The cross-section of the coating layer with the substrate was initially examined by an optical microscope. Figure 3a,b shows the multiple layers of NiTi coatings by plasma spraying samples 1 and 2 on the top of the substrate. The samples were cut from the substrate with a remaining thickness of 0.1 mm substrate. The samples were mounted in conductive Bakelite through the thickness. Both samples showed the multi-layers of coating on the substrate. The interface between steel and the coating layer showed better adhesion without any porosity. Various coating layers with adjoining areas are marked as dark regions (Figure 3a,b). Sample 2 also showed good adhesion of substrate steel with coating layers. However, there were some impurities observed in sample 1 that arose from the contamination from sample preparation. 

**Figure 4:**
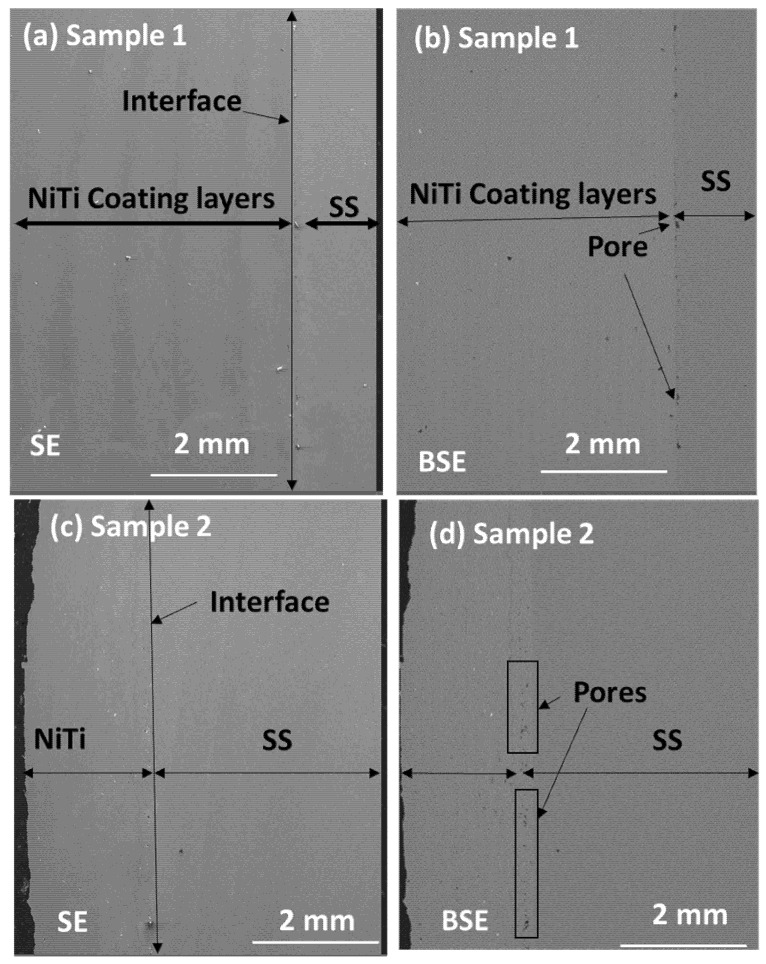
(**a**–**d**) SE and BSE mode of samples 1 and 2 with substrate stainless steel showing minor porosity at the interface of sample 1 and major porosity at the interface of sample 2.

To examine the interface and the coating layers, samples were investigated using both SE and BSE modes in SEM. Figure 4a–d represents the SE and BSE mode of images of samples 1 and 2 with the substrate stainless steel. In SE mode, the interface joining of the substrate stainless steel and coating layers showed good joining areas; however, BSE mode revealed more clarity in the interface zone. Sample 1 revealed slight porosity in certain areas; however, there was more porosity in the interface regions of sample 2. In this case, sample 1 showed a better structure of samples within interlayers of the coating region. The joining between coating layers and the substrate showed better adhesion in sample 1 (Figure 4c); however, sample 2 showed internal porosity in various regions of multilayers of coating areas (Figure 4d). 

**Figure 5:**
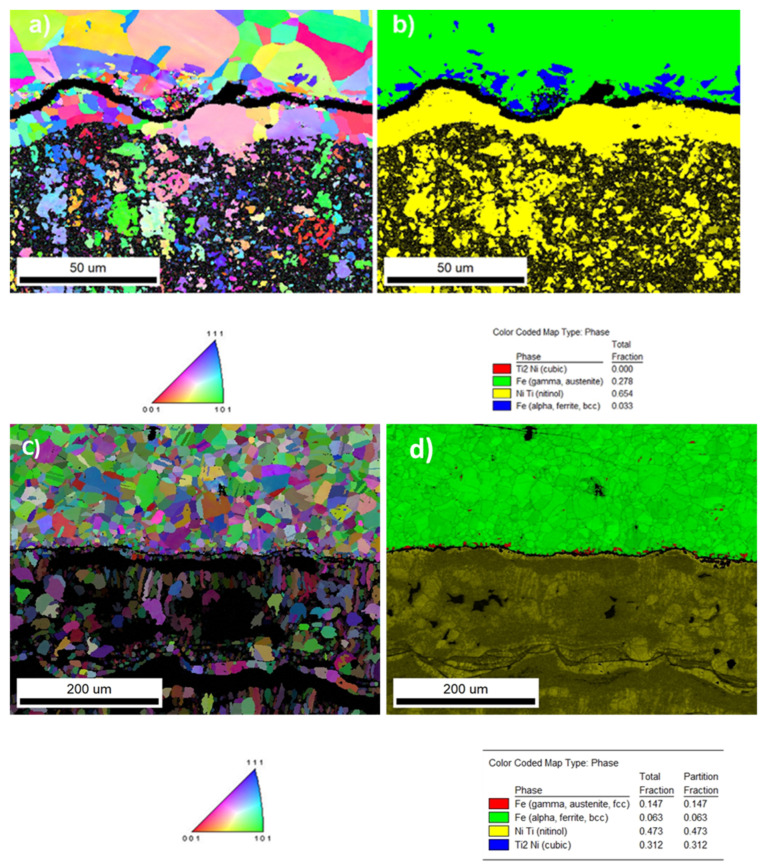
(**a**,**b**) EBSD image pattern of the interface region. (**a**) An orientation map of the inverse pole figure (IPF) map overlayed on a greyscale confidence index (CI). Colorized legend of phases is given below—the scale was the same for all presented phases. (**b**) The phase composition overlayed on a greyscale confidence index again. The fraction of phases is given below the map with the color code (sample 1). (**c**,**d**) EBSD image of sample 2 showing the IPF figure and phase composition.

An electron back-scattered diffraction (EBSD) investigation was carried out on sample 1 at the interface of the region. Phases of NiTi and Fe were revealed as the austenite and ferrite shown in the color code map. Figure 5a–d represents the EBSD maps of samples 1 and 2.

**Figure 6:**
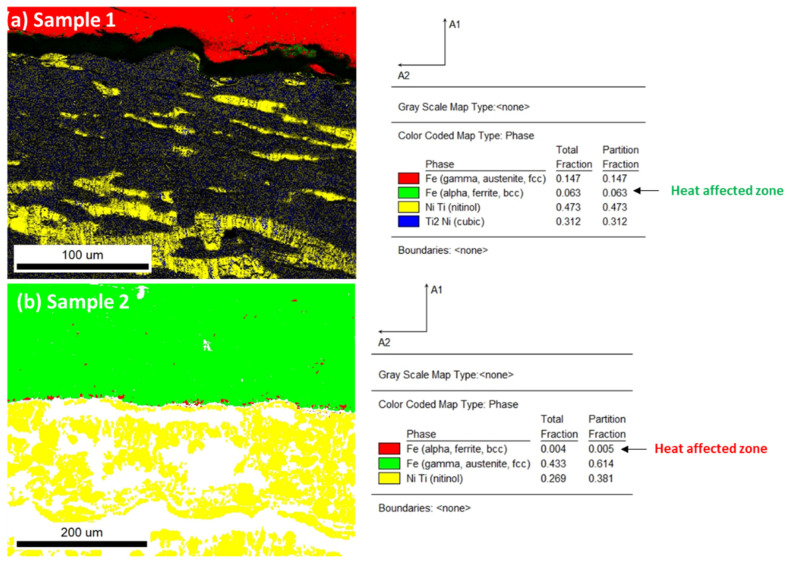
(**a**,**b**) The interface region for samples 1 and 2. A very minor region of Fe, alpha, and ferrite was located along the interface. (**a**) Sample 1, alpha, ferrite, total fraction, 0.063; (**b**) sample 2, alpha, ferrite 0.004 from the total fraction. Only points with CI > 0.1 are presented.

The heat-affected zone on the substrate of the samples along the cross-section of the coating from the substrate surface was examined by EBSD analysis. It was observed that ferrite appeared in the heat-affected zone, with non-uniform regions with smaller grains formed along the interface in the substrate with a maximum of 10 µm thickness. The region of the heat-affected zone in both samples is shown in Figure 6a,b.

**Figure 7:**
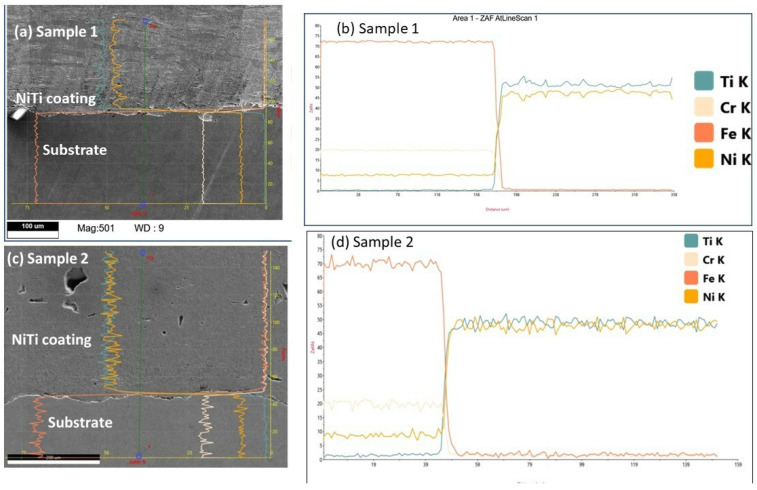
(**a**,**b**) Line analysis for sample 1 with elemental diffusion for Ti, Cr, Fe, and Ni K spectra lines. (**c**,**d**) Region of line analysis for sample 2. (**d**) Elemental diffusion of spectra lines Ti, Cr, Fe, Ni, and K.

The microstructural changes along the heat-affected zone were examined by the line scan analysis. To understand the phase changes that led to the diffusion of elemental composition, phase change, and microstructural investigation, line analysis is presented in Figure 7a,d. The results of line analysis show that there was no diffusion of elements from the substrate to the coating region. The spectra from the Fe lines reached a minimum value in the coating region; however, the lines of Ni and Ti achieved maximum values. This confirms that there was no diffusion of elements from the substrate to the coating layers for both samples. There was a homogenous distribution of elemental composition in the NiTi coating layers (provided as Supplementary Files). 

### 3.2. Thermal Characterization of Plasma-Sprayed NiTi Samples

**Figure 8:**
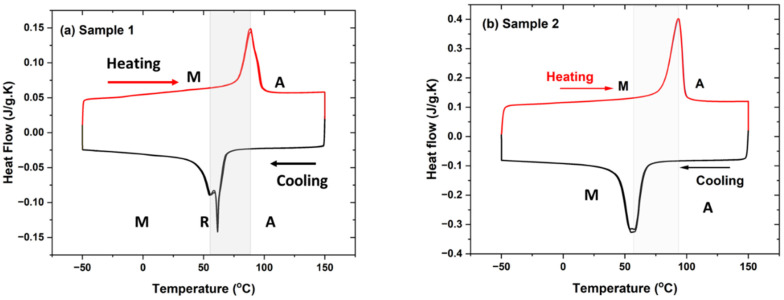
(**a**,**b**) DSC images of samples 1 and 2 showing transformation temperature during cooling and thermal cycles for samples 1 and 2. Sample 1 shows austenite transformation from R-phase to the martensite phase; however, R-phase was absent in sample 2.

Thermal characterization of plasma-sprayed samples 1 and 2 is represented in Figure 8a,b. The transformation temperature of samples was observed in cooling and heating cycles. Sample 1 showed the transformation temperature of phases at the start of the R-phase temperature at 71 °C, with R-phase finish temperature at 59 °C, and with R phase peak at 62 °C. The second peak of the martensite phase started at 59 °C, with the martensite peak at 55 °C, with a finish temperature of 33 °C in cooling mode. The heating cycle showed the transformation of martensite to austenite phases. Austenite started at a temperature of 64 °C, followed by an austenite peak of 88 °C, and ended at an austenite finish temperature of 101 °C. Sample 2 shows the transformation of austenite to martensite phase from the cooling cycles and back to the austenite phase during the heating cycle. The transformation temperature peak of martensite started at a temperature at 69 °C with a peak temperature of 57 °C and a finish temperature of 38 °C. The transformation of austenite started at a temperature of 62 °C with a peak temperature of 93 °C and with a finish temperature of 103 °C. The measurement was conducted at least twice for both samples, with it observed that both peaks coincided. The initial NiTi powder used for spraying showed the phases of the austenite phase (start: 43 °C, peak: 52 °C, finish: 62 °C) and martensite phase (start: −23 °C, peak: −33 °C, finish: −43 °C) during thermal cycles. The transformation temperatures of the plasma-sprayed samples shifted towards a higher value than feedstock powder that may arise from various melting zones from the plasma arc chamber, varying chemical composition, the presence of intermetallic phases, the internal porosity region, or accumulated residual stress in the samples. 

### 3.3. Phases Analysis of Plasma-Sprayed Samples

**Figure 9:**
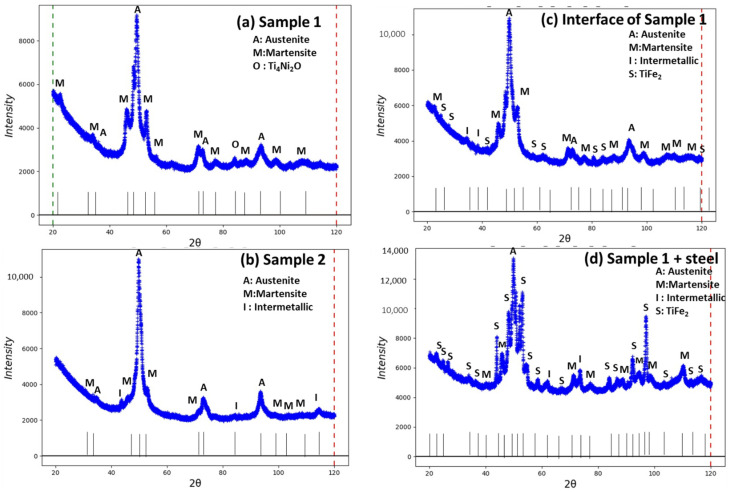
XRD pattern of various phases in plasma-sprayed samples 1 and 2 (**a**,**b**) and the interface of sample 1 and sample 1 with stainless steel as substrate (**c**,**d**).

Figure 9a,b represents the XRD peaks of sample 1, sample 2, and two more samples from the interface and with the substrate of sample 1. Both samples show the presence of various phases of the Ni-Ti system such as the austenite cubic phase, martensite, monoclinic, and R-phase (pre-martensite phase) with a hexagonal structure, intermetallic, metastable phase of Ti_3_Ni_4_, and oxide phases of Ti_4_Ni_2_O. Table 2 represents the quantitative volume fraction of various phases for standalone plasma-sprayed samples 1 and 2 separated from the substrate, then the additional sample 1 considered with the substrate as a base (both the upper coating and lower side steel). Sample 1 contained the austenite phase of 48.0 wt %, martensite of 39.9 wt %, R-phase (pre-martensite or austenite phase) of 8.8%, and a minor amount of oxide phase Ti_4_Ni_2_O of 3.2 wt % (Figure 7a). However, sample 2 contained phases of austenite of 43.2 wt % and martensite of 25.8 wt %, with some intermetallic phases of Ti_3_Ni_4_ of 30.6 wt %, without R and oxide phases (Figure 7b). Sample 1 with substrate steel (0.1 mm) was investigated on the upper and lower sides for accurate analysis of interface phases. Sample 1 with the interface from the upper surface from the coating layer showed the presence of stainless steel as a base with a phase composition of austenite of 37.5 wt %, martensite of 24.3 wt %, R-phase of 18.9 wt %, intermetallic phase of Ti_3_Ni_4_ of 6.5 wt %, and TiFe_2_ of 12.9 wt % (Figure 9c). Sample 1 with lower surface steel showed the presence of TiFe_2_ as a major phase with some amounts of austenite, martensite, and intermetallic phases (Figure 9d). The quantitative volume fraction of phases is estimated in Table 2. The interface contained less diffusion of stainless steel, and more on NiTi phases from plasma coating sides. However, the sample with steel as substrate showed major phases of steel with minor phases of NiTi diffusion from coating to base. 

### 3.4. Thermo-Mechanical Characterization of Plasma-Sprayed Coatings

**Figure 10:**
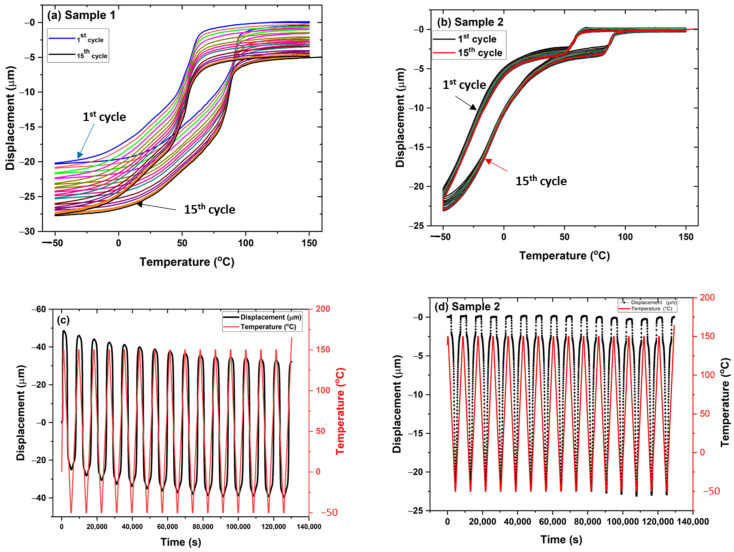
(**a**–**d**) Thermo-mechanical analysis of samples 1 and 2 for 15 cycles showing displacement versus temperature (**a**,**b**), the first cycle corresponds to a blue color with follow up subsequent cycles until 15th cycle (black color) for sample 1 (**a**), the first cycle corresponds to black color with follow up subsequent cycles until 15th cycle (red color) for sample 1 (**b**) and displacement and temperature versus time (**c**,**d**).

**Figure 11:**
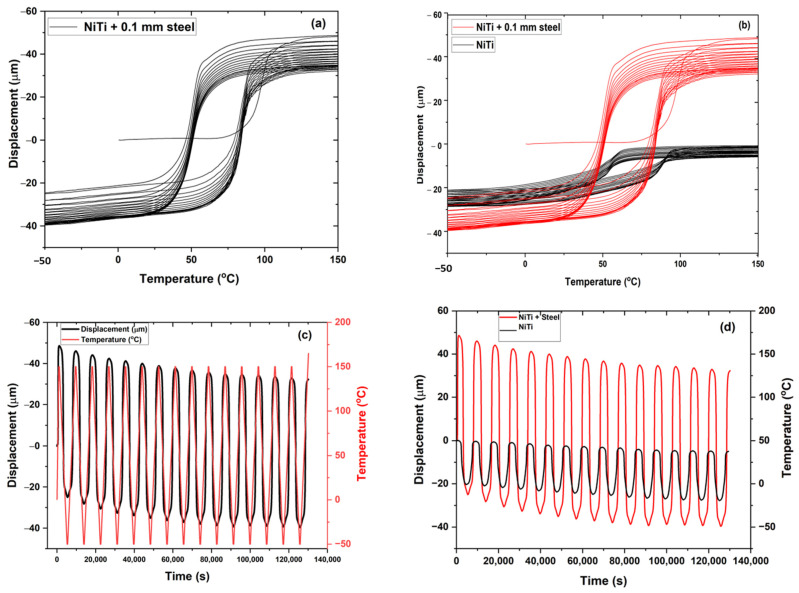
(**a**,**b**) Sample 1 with 0.1 mm of steel and a comparison between sample 1 and sample 1 with 0.1 mm of steel; (**c**,**d**) displacement versus time graph for (**a**,**b**).

Figure 10a,b represents the thermo-mechanical behavior of samples 1 and 2 in the presence of a static load of 100 mN under thermal cycles. The results indicate the deformation of samples as the function of temperature from −50 °C to 150 °C. The multiple cycles were carried out on the samples, with 15 cycles for both samples. It was observed that during each cycle there was residual strain accumulated in the sample. Finally, the sample returned to the position from the initial position with a displacement of −7.5 µm for sample 1 and −2 µm for sample 2. However, the hysteresis of sample 2 showed irregular behavior compared to sample 1 with two conjugated areas of cycles. This may have been due to inhomogeneities of elemental composition along the coating layers, or the distribution of irregular internal stress developed in the samples during thermal cycles. Figure 10c,d plots the sample displacement’s behavior as the temperature and time function. It was observed that sample 1 showed a subsequent increase in displacement as the function of time with a fixed temperature cycle from the cooling to the heating range. However, in sample 2, the displacement was not so prominent compared to sample 1. The sample returned to its original position after heating from bending deformation. Sample 2 had better recovery of the displacement to the original position after heating with a change in position of −2 µm from the original position. Figure 11a–d shows sample 1 with substrate steel (0.1 mm as the base) under a thermo-mechanical cycle. The sample contained two layers of composition with upper NiTi as the coating layer deposited by plasma spraying and lower stainless steel as the substrate. The sample showed a significant reduction in displacement position with an accumulation of internal residual stress from the two layers’ deformation during thermal cycles. The plastic strain deposited in the sample showed a reduction in the displacement of −90 µm from the original position (Figure 11a). The two samples were compared without substrate and with the substrate for sample 1 under static load as the function of the thermal cycle. The change in displacement was significantly large for sample 1 with the substrate. Figure 11c,d shows the graph that represents displacement versus time. The sample with substrate accumulates residual stress in the sample with each cycle. There was a significant change in the sample’s behavior that may arise from the interface joining of the elastic–plastic region of material from steel and NiTi.

### 3.5. Hardness of the Plasma-Sprayed Sample 

**Figure 12:**
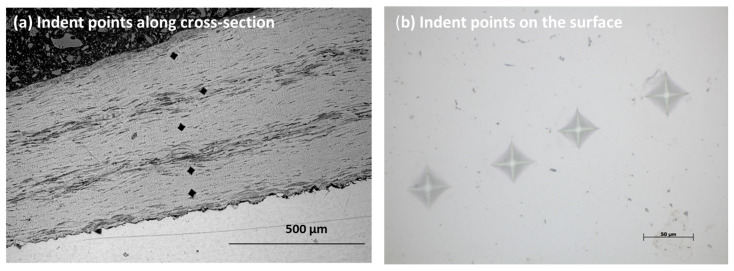
(**a**) indent points along the cross-section; and (**b**) indent points on the surface of the plasma-sprayed coatings.

Figure 12a–d displays the hardness evaluation along the cross-section of the sample. At least five points were considered for the average values of the hardness test [25,26]. The load of 1.961 N was used for 10 s to measure the indent points on the surface and cross-section of the sample. The image of indent points along the cross-section and surface of the sample is shown in Figure 12a,b. Table 3 represents the hardness of both samples on the surface and cross-section. There was a slight difference in the values of hardness at the surface and the cross-section. The microhardness results show from coating to substrate. Increased hardness was shown at the surface compared to the cross-section region for NiTi coating samples with lower hardness for the substrate [27,28]. 

The value of 230–277 HV corresponded to the hardness of NiTi prepared by plasma spraying. The values matched well with other researchers’ findings for NiTi SMA alloy prepared by plasma spraying [29]. The lower value of hardness 231–234 HV corresponded to the B19 martensite phase of NiTi, whereas the higher value corresponded to the austenite phase of the NiTi coating structure [30]. Similarly, the hardness of the stainless steel matched well with the austenite phase values of 129 HV. As the ferrite phase formed in a minor amount near the interface in the heat-affected zone, it was hardly able to contribute towards the hardness of the core substrate. 

## 4. Conclusions

The NiTi shape memory alloy formed a thick coating layer on the stainless steel by the plasma spraying method. The effective coating layer of 500 µm was obtained at 12 kW power with a feed rate of 4.2 g·min^−1^. The better interfacial adhesion of the coating layer with the substrate stainless steel was observed without any porosity. The plasma-sprayed sample showed austenite and martensite, as well as a minor oxide compound. The grain size distribution along the interface showed the distribution of phases that belong to the NiTi austenite phase and substrate stainless phases of Fe and austenite. The hysteresis effect of the standalone sample showed the behavior of the shape memory effect. There was a larger hysteresis effect in the shape memory effect with samples with coating and substrate as material. However, there was a large accumulation of plastic strain in the material with multiple cycles. The change in displacement was more prominent in the composite material compared to NiTi coating. The hardness of the NiTi coating and the substrate confirmed the intermetallic alloy and substrate. 

## Figures and Tables

**Table 1:** Parameters for sample preparation. Chemical composition of powder and substrate.

Sample	Plasma Gas (Ar + H_2_)	Substrate	Plasma Torch Power (kW)	Feeding Rate (g/min)	Spraying	Net Powder Spray Time (s)	Comment
Sample 1	10 + 2	Stainless steel with grit blasting	12	4.2	30 × 6	180	Good
Sample 2	10 + 1	Stainless steel	9	2.1	25 × 6	150	Thin coating layer
Initial material	Particle size	Chemical composition, at (%)					
Stainless steel	-	0.11% N	17.5–19.5% Cr	8–10.5% Ni	0.05% P	0.07% C	1% Si
NiTi powder	20–60 µm			50	50		

**Table 2:** Various phases of the Ni-Ti system in plasma-sprayed samples 1 and 2.

Phases	Crystal System	Sample 1 Semi-Quant% (*w*/*w*)	Sample 2	Sample 1 with Interface	Sample 1 (Lower Surface) Steel
Austenite	Cubic—Pm-3m (01-076-3614)	48	43.2	37.5	6.0
Martensite	Monoclinic, P21/m (01-078-2550)	39.9	25.8	24.3	22.7
R-phase	Hexagonal, P-3 (01-075-0878) (01-078-4620)	- 8.8	- -	5.6 13.3	8.1 7.0
Intermetallic Ti_3_Ni_4_	Rhombohedral, R-3 (04-001-1903)	-	30.6	6.5	23.2
TiFe_2_	Hexagonal, P63/mmc (04-004-6664)	-	-	12.9	33.1
Ti_4_Ni_2_O	Cubic, Fd-3m (04-011-8824)	3.2	-	-	-

**Table 3:** The hardness of the plasma-sprayed sample at the surface and cross-section from center and edge regions.

Sample (Force (N): 1.961, Time: 10 s)		Surface (HV)		Cross-Section (HV)	
Sample 1					
At center	At edge	238.6 ± 18.6	264	231.0 ± 15.9	-
Sample 2					
At center	At edge	277.0 ± 19.4	274	259.8	-
Steel (substrate)		129		129	

## Data Availability

Data will be available on request.

## Supplementary Materials

The following supporting information can be downloaded at: https://www.mdpi.com/article/10.3390/ma15238598/s1.
