# Supplementary material for: Interfacial Adhesion of Thick NiTi Coating on Substrate Stainless Steel
Source: Materials (Basel). 2022 Dec 2;15(23):8598. doi: 10.3390/ma15238598 (PMC9738793; doi:10.3390/ma15238598)
Supplement: Supplementary file 1 [file materials-15-08598-s001.zip › materials-2035147-supplementary.pdf]

Supplementary files

Sample 1 : elemental composition

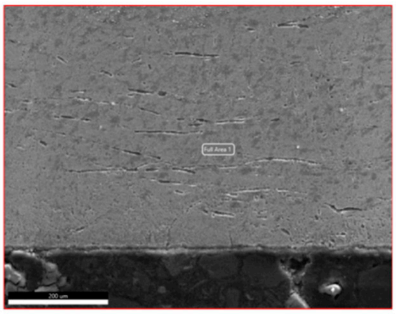

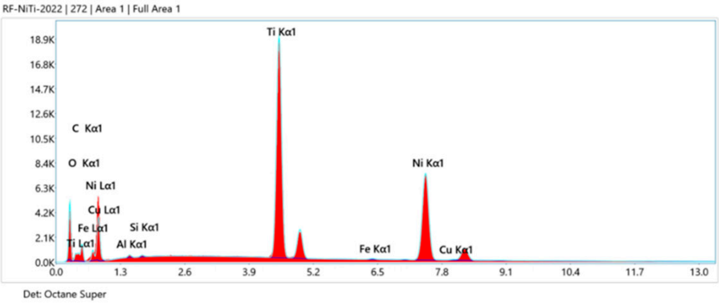

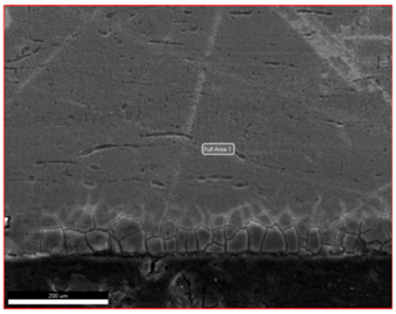

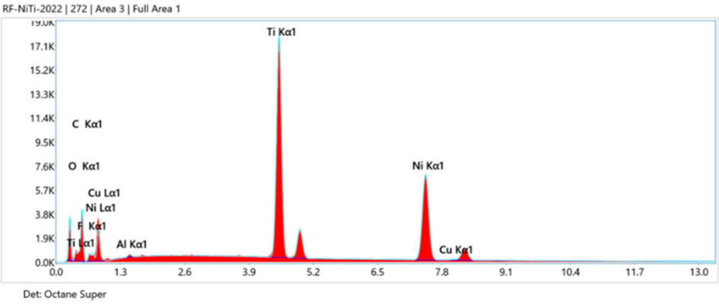

Sample 1: Line analysis

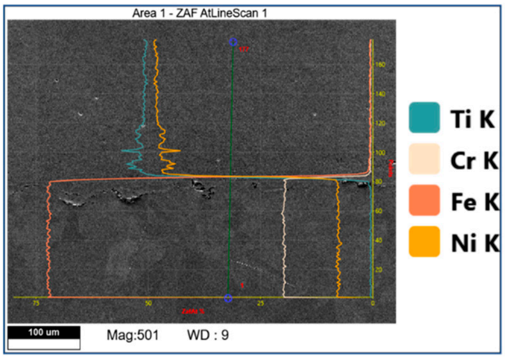

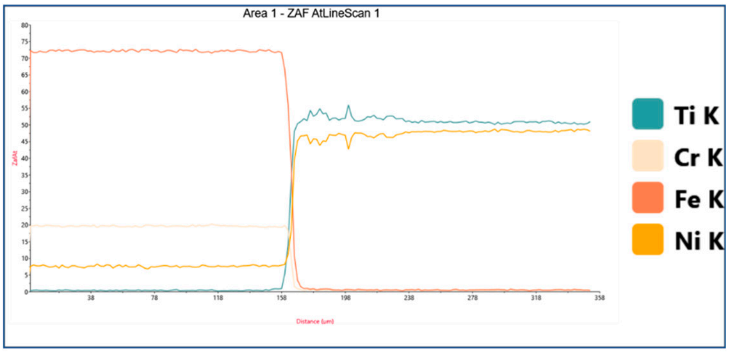

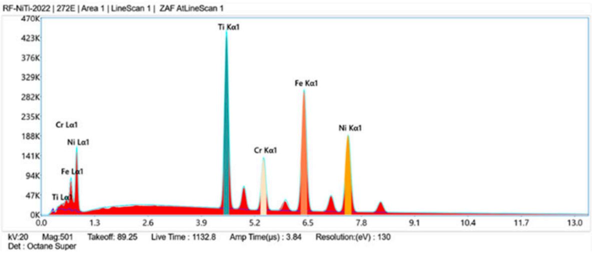

eZAF Quant Result - Analysis Uncertainty: 3.09 %

Element	Weight %	MDL	Atomic %	Error %	Net Int.	R	A	F
Ti K	24.4	0.02	27.6	2.8	4629.9	0.8831	0.8909	1.0869
Cr K	9.9	0.02	10.3	3.3	1475.0	0.8927	0.8638	1.1265
Fe K	32.7	0.03	31.7	2.9	3680.1	0.9028	0.8925	1.0849
Ni K	32.9	0.04	30.4	3.0	2511.2	0.9136	0.8780	1.0360

Sample 2 : Line analysis

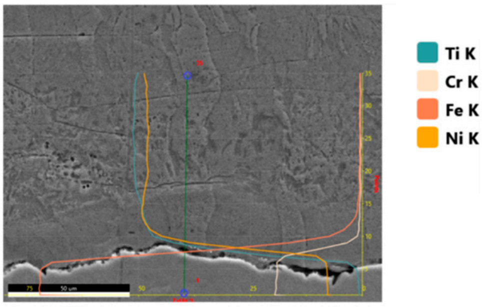

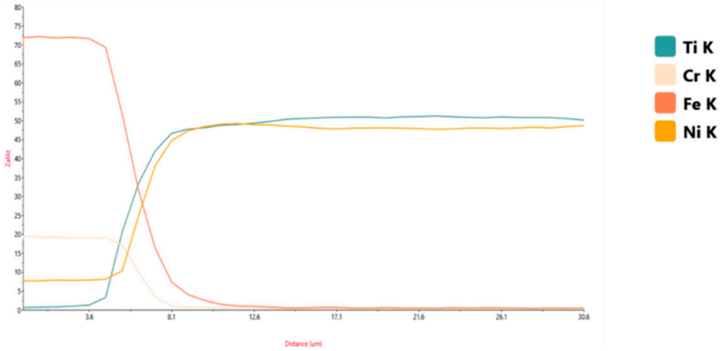

Composition

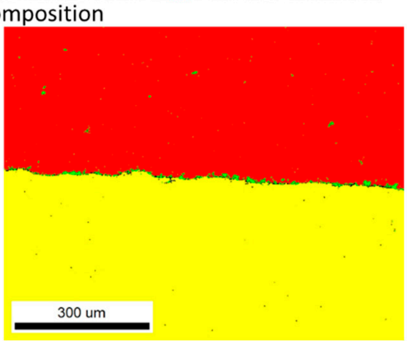

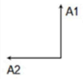

Gray Scale Map Type:<none>

Color Coded Map Type: Phase

Phase	Total Fraction	Partition Fraction
Fe (gamma, austenite, fcc)	0.480	0.480
Fe (alpha, ferrite, bcc)	0.006	0.006
Ni Ti (nitinol)	0.512	0.512

Boundaries: <none>
